# Infrastructure for Sustainable Protein Innovation: A Global Value Chain Framework for CDMOs in Fermentation-Based Biomanufacturing

**DOI:** 10.3390/foods15081341

**Published:** 2026-04-13

**Authors:** Germano Glufke Reis, Antonella Samoggia, Maria Clara Manzoki

**Affiliations:** 1 SFoodLab—Sustainable Food Value Chains Laboratory, School of Management, Federal University of Parana, Curitiba 80210-170, Parana, Brazil; glufkereis@ufpr.br; 2 Department of Agricultural and Food Sciences, University of Bologna, Viale Fanin 50, 40137 Bologna, Italy; 3 Interdepartmental Centre for Industrial Agri-Food Research (CIRI-AGRO), Alma Mater Studiorum, Università di Bologna, Via Quinto Bucci 336, 47521 Cesena, Italy; 4 Bioprocesses Engineering and Biotechnology Department, Federal University of Parana, Curitiba 80210-170, Parana, Brazil; mariamanzokiii@gmail.com

**Keywords:** fermentation-based proteins, contract development and manufacturing organizations, global value chains, innovation governance, sustainable food systems, alternative protein scaling, biomanufacturing, circular production

## Abstract

Achieving more sustainable production in emerging biomanufacturing sectors depends not only on technological innovation but also on how production systems are organized, governed, and scaled. Fermentation-derived proteins produced through biomass and precision fermentation offer promising pathways to reduce the environmental impacts of conventional livestock production. However, their sustainability and circularity outcomes depend heavily on access to biomanufacturing infrastructure and coordination along global value chains. Drawing on Global Value Chain (GVC) theory and an integrative review of more than 40 academic and industry sources published between 2017 and 2026, spanning global value chain governance, biomanufacturing scale-up, CDMO functions, and sustainability and bioeconomy transitions, this study develops a conceptual framework that positions Contract Development and Manufacturing Organizations (CDMOs) as key infrastructural intermediaries in fermentation-based protein systems. CDMOs facilitate access to fermentation capacity, technical expertise, and regulatory capabilities, thereby shaping governance arrangements, capability development, and the scaling of innovation. In doing so, they influence how cleaner production principles, such as resource efficiency, circular feedstock integration, and improved environmental performance, are translated into industrial practice. The analysis also highlights risks linked to CDMO-driven scaling, including infrastructure concentration, dependency dynamics, and unequal access across regions. By integrating GVC perspectives with insights from sustainability transitions and the circular bioeconomy, the article advances understanding of how infrastructural intermediaries shape cleaner production outcomes in emerging biomanufacturing value chains.

## 1. Introduction

Protein production from more sustainable sources has gained increasing attention from industry stakeholders and policymakers, driven by advances in alternative protein technologies such as precision fermentation and their implications for food safety, regulation, and innovation, alongside their potential to mitigate environmental impacts and foster inclusive economic development [1]. Conventional livestock production remains one of the most environmentally intensive activities in the agri-food sector, driving deforestation, biodiversity loss, freshwater depletion, and greenhouse gas (GHG) emissions [2,3]. Even under efficient systems, beef production emits approximately 25 kg of CO_2_ per 100 g of protein, while less efficient operations can reach up to 105 kg CO_2_ per 100 g, making animal-based meat among the highest-emitting food products globally.

In contrast, alternative protein sources, particularly plant-based and fermentation-based proteins, require significantly less land, water, and energy, and are associated with substantially lower GHG emissions [4,5,6]. However, the realization of these environmental advantages depends not only on technological characteristics, but also on how production systems are organized, scaled, and coordinated across value chains [7,8,9].

Fermentation-based proteins have emerged as a particularly promising domain for advancing cleaner production and supporting the transition toward a low-carbon, circular bioeconomy. Recent studies highlight the growing relevance of both biomass and precision fermentation as alternatives to animal protein, emphasizing their distinct technological characteristics and sustainability potential [10,11]. Yet, their contribution to cleaner production is not inherent to the technology alone. It depends on production scale, access to food-grade infrastructure, and the ability to coordinate activities across different stages of production. The EAT–Lancet Commission [12] underscores the need for systemic transformations in food systems to remain within planetary boundaries, reinforcing the importance of how these technologies are implemented in practice.

Fermentation pathways can contribute to circular bioeconomy strategies when they enable the use of agro-industrial side streams and by-products as inputs for food-relevant biomasses and ingredients [13]. Evidence from the bio-residual valorization literature suggests that resource-efficiency gains depend on viable configurations across procurement, processing, and market formation, as well as on supportive governance and regulatory conditions [14]. Whether such benefits materialize at scale therefore depends on how fermentation-based production systems are industrialized and coordinated.

As the bioeconomy evolves toward more structured industrial configurations, the ability to scale and organize production systems becomes central [15,16]. Fermentation-based protein systems provide a relevant context to examine how technological capabilities, value creation, and sustainability outcomes are distributed across actors and regions [17], particularly in light of circular supply chain governance approaches that aim to minimize waste and enhance resource efficiency across production networks [18,19]. However, realizing this potential remains constrained by capital intensity, regulatory complexity, and the limited availability of food-grade biomanufacturing infrastructure [20,21].

In this context, fermentation-based production systems require extensive scale-up efforts, including the transition from laboratory experiments to pilot-scale operations and the production of batches under industrially relevant conditions. Pilot-scale production plays a critical role in validating both technical feasibility and economic assumptions, which are essential for reducing uncertainty and attracting investment. Scale-up failures remain common due to non-linear effects in mass transfer, oxygen limitation, and downstream recovery performance [22]. At the same time, the equipment and expertise required for scale-up are highly specialized and capital-intensive, often exceeding the capabilities of early-stage firms.

Against this backdrop, Contract Development and Manufacturing Organizations (CDMOs) have emerged as increasingly relevant intermediaries in the scale-up of fermentation-based production. Traditionally associated with the pharmaceutical sector, CDMOs are expanding into food and bio-based applications as part of broader changes in biomanufacturing. Existing studies highlight their role in providing access to fermentation capacity, bioprocess development, and regulatory support, as well as their influence on scale-up pathways and investment decisions [23]. This raises important questions about how access to infrastructure shapes the development of fermentation-based protein systems.

At the same time, the implications of CDMO-driven scaling remain ambiguous. Analyses of bioeconomy transitions show that sustainability outcomes are transmitted through global production networks, often generating uneven regional effects and trade-offs between competitiveness and food system resilience [24]. In Latin America, fermentation-based proteins are often framed as opportunities for sustainable industrial development, yet their advancement remains constrained by infrastructure gaps and fragmented innovation systems [25]. In parallel, initiatives such as the Biomes Program in Brazil illustrate how fermentation-based approaches can support more territorially embedded and regenerative bioeconomy strategies [26]. These dynamics highlight both the opportunities and the structural constraints shaping cleaner production in emerging contexts, particularly where infrastructure, regulatory coordination, and industrial support systems remain uneven.

Although prior studies have addressed fermentation-based proteins, scale-up bottlenecks, CDMO roles in biomanufacturing, and governance in global value chains, these discussions have largely evolved in parallel. This study connects these strands by framing CDMOs as infrastructural intermediaries that influence governance patterns, upgrading trajectories, and cleaner production outcomes in fermentation-based protein value chains. In doing so, it shows how access to external biomanufacturing capabilities shapes not only the ability to scale, but also how sustainability and circularity ambitions are translated into production practice.

This article focuses on biomass and precision fermentation as two segments with distinct technological and value chain characteristics compared to plant-based or cell-based alternatives [10,13,27]. It adopts a conceptual, theory-building approach to examine how CDMOs shape cleaner production outcomes, technological trajectories, and development dynamics in fermentation-based protein systems. The objective is to develop an integrative framework that positions CDMOs as enabling structures, potential bottlenecks, and coordination nodes in emerging biomanufacturing ecosystems. By doing so, the article contributes to debates on cleaner production, circularity, and the role of infrastructure in sustainable innovation.

More specifically, it addresses a gap at the intersection of GVC analysis, CDMO studies, and cleaner production by conceptualizing CDMOs not merely as service providers, but as structures that shape governance, upgrading, and cleaner production dynamics in fermentation-based biomanufacturing systems. While these dimensions have been examined separately, their interaction, particularly the role of CDMOs in mediating access to scale-up infrastructure, has not been systematically addressed in prior work.

## 2. Theoretical Background

### 2.1. GVC Theory

GVC theory provides a robust analytical framework for understanding how value is created, captured, and distributed across firms and geographies within increasingly fragmented and globally dispersed production systems. Developed in the 1990s within development studies and international political economy [28], the GVC approach has since expanded to incorporate insights from economic geography, international business, and innovation studies. Its evolution has made it especially suitable for examining the organization of innovation, the distribution of capabilities, and the institutional and environmental dynamics of emerging industries [29,30].

The theory identifies five inter-related analytical dimensions: input–output structure, geographical scope, governance mechanisms, upgrading, and institutional context [30,31]. These dimensions are essential for analyzing how value chains operate and evolve, especially in sectors where technological complexity, sustainability goals, and new forms of inter-firm coordination play a central role.

The input–output structure maps the sequence of activities from research and development (R&D) and design to production, marketing, and distribution that define the flow of tangible and intangible value. It clarifies who does what in the chain and how different actors interact across stages of value creation [30]. The geographical scope dimension examines how these activities are distributed spatially, often reflecting global asymmetries in capabilities, infrastructure, and institutional quality [32].

Governance refers to how value chains are coordinated and controlled, particularly through relationships between lead firms and suppliers. Gereffi et al. [7] identify five governance types—market, modular, relational, captive, and hierarchy—each defined by the complexity of transactions, codifiability of information, and supplier capabilities. These structures determine how information flows, how power is exercised, and how responsibilities and risks are distributed along the chain.

Upgrading is a core concept for understanding how firms and regions improve their competitive position within the chain. Humphrey and Schmitz [8] distinguish between process, product, functional, and intersectoral upgrading. The ability to upgrade depends not only on internal capabilities but also on access to knowledge, infrastructure, and enabling institutions [9,32].

Finally, the institutional context shapes the rules of the game, including intellectual property regimes, quality standards, public policies, and industrial strategies. Effective governance of global value chains requires coordinated efforts from both private and public actors to ensure innovation, environmental compliance, and equitable value capture [33].

Applying GVC theory to fermentation-based protein production within the broader context of the bioeconomy enables a nuanced understanding of how sustainability, innovation, and industrial scaling interact in emerging bioindustrial systems. In this study, the bioeconomy is understood as the reconfiguration of production systems to rely on renewable biological resources, knowledge-intensive processes, and circular value chains [16,34]. This framing aligns with recent literature emphasizing the need to build robust, sustainable value chains that connect innovation niches with industrial production systems [35].

Recent GVC-based analyses further emphasize that the transition from innovation-oriented activities to industrial production is rarely linear. In agri-food systems, incumbent firms and existing industry structures play a decisive role in shaping which technologies scale, how they scale, and where bottlenecks persist along value chains [36]. In parallel, advancing the bioeconomy requires overcoming fragmentation and creating industrial infrastructures that support scale, efficiency, and environmental goals [37].

CDMOs play a key role in this transformation by enabling firms to access critical capabilities, infrastructure, and regulatory expertise. Their integration into global value chains may facilitate upgrading, reduce entry barriers, and redistribute capabilities. At the same time, however, this intermediation introduces new dependencies and coordination challenges that must be understood in light of institutional and geographical asymmetries.

This GVC-based perspective is particularly relevant in the context of sustainability transitions. As D’Amato et al. [16] emphasize, achieving green, circular, and bioeconomy goals requires rethinking how production systems are designed, governed, and integrated with social and ecological systems. GVC theory contributes to this agenda by offering tools to analyze not only the economic organization of value chains, but also their institutional embeddedness and potential to deliver cleaner and more inclusive production outcomes.

Despite its analytical strengths, GVC theory presents limitations when applied to emerging, innovation-intensive sectors. The framework has been primarily developed to explain governance structures and inter-firm coordination [29,30], while offering more limited treatment of technological uncertainty, early-stage innovation dynamics, and non-linear scaling processes. In the context of fermentation-based biomanufacturing, where technological feasibility, regulatory pathways, and infrastructure constraints evolve simultaneously, these aspects are particularly salient. Nevertheless, GVC remains an appropriate analytical lens for this study, as it provides robust tools to examine how access to infrastructure, governance configurations, and upgrading opportunities are distributed across actors and regions, which are central concerns in the scale-up of alternative protein systems.

### 2.2. CDMOs in Biomanufacturing and Biotechnology: Development, Scale-Up, Regulatory Support, and Manufacturing

CDMOs play an increasingly strategic role in biomanufacturing and biotechnology by offering vertically integrated services that span early-stage development to commercial-scale production. Their presence is significant across sectors such as pharmaceuticals, agriculture, food and beverages, cosmetics, and industrial biotechnology, where they provide process design, scale-up, regulatory support, and Good Manufacturing Practices (GMP)-compliant manufacturing capabilities [38,39]. In early-stage development, CDMOs support clients through upstream and downstream process optimization, scale-up, product formulation, stability testing, and technology transfer, all of which require high technical precision to ensure product quality and regulatory compliance.

While the pharmaceutical sector has traditionally been the core domain for CDMOs, the emergence of complex bio-based innovations, such as cellular agriculture, has expanded their relevance. These new domains require the integration of process development, quality assurance, and scalable production infrastructures. Bioprocess development typically advances through laboratory, pilot, and industrial scales, with each phase requiring dedicated equipment, protocols, and biosafety standards [40]. CDMOs facilitate this transition by reducing capital intensity, time to market, and operational risk for innovation-driven firms.

Unlike traditional suppliers or contract manufacturers (CMOs), CDMOs integrate upstream R&D, regulatory strategy, and downstream manufacturing within a unified operational model [38]. This positions them as critical intermediation platforms between scientific innovation and industrial deployment. Building on earlier evidence of functional fragmentation and specialized intermediaries in pharmaceutical value chains [41], recent analyses show that CDMOs have evolved into integrated platforms combining upstream R&D, regulatory strategy, and downstream manufacturing [38]. Evidence from biopharmaceutical value chains shows that CDMOs evolve from pure manufacturing providers into integrated technology platforms, progressively internalizing upstream development and regulatory capabilities as production complexity and scale-up risks increase [42].

In the pharmaceutical global value chain, CDMOs operate as globally distributed, knowledge-intensive actors embedded in innovation ecosystems [43]. Biomanufacturing of biologics, in particular, relies on such infrastructures due to the complexity of microbial and cellular processes, which cannot be replicated through synthetic chemistry alone.

While CDMOs are often associated with enabling circular production models, it is important to distinguish between the origin of circular strategies and their operationalization. In most cases, decisions regarding the use of agricultural residues or alternative feedstocks are defined upstream by researchers, startups, or client firms, rather than by CDMOs themselves. CDMOs primarily provide the technical, infrastructural, and regulatory conditions required to implement these strategies at scale, including process adaptation, quality assurance, and coordination with feedstock suppliers. Their GMP-compliant infrastructure and flexible production capacity are particularly relevant for startups and small enterprises, which often face barriers to entry due to a lack of capital and limited access to pilot, food-grade fermentation facilities. The growth of the biopharmaceutical CDMO market, valued at USD 40.1 billion in 2024 and projected to grow at a compound annual growth rate (CAGR) of 11.1% through 2030, reflects a broader shift toward outsourcing in response to increasing regulatory and technological complexity [44].

While quantitative estimates for the biopharmaceutical CDMO market are well established, comparable market data for food-grade CDMOs in fermentation-based protein systems remain limited. Existing industry reports on alternative proteins emphasize the rapid growth of fermentation ventures, infrastructure expansion, and increasing investment flows, but do not yet provide consolidated estimates of the size and structure of the food-grade CDMO segment. This absence reflects the early-stage and fragmented nature of the sector, where market boundaries and outsourcing structures are still in formation, and biomanufacturing capacity is often embedded within hybrid organizational models rather than clearly defined outsourcing markets. In this context, evidence from the pharmaceutical sector is used as an indicative proxy to illustrate broader outsourcing dynamics, while acknowledging important sectoral differences in margins, regulatory requirements, and scale-up pathways [20,21,27].

### 2.3. Why the Alternative Protein Sector Is Unique

While the alternative protein sector shares certain infrastructure, regulatory, and technological demands with pharmaceutical biomanufacturing, it presents unique challenges and opportunities that require sector-specific analytical attention. Products derived from precision and biomass fermentation must not only comply with food safety standards but also meet complex sensory, nutritional, and cultural expectations from consumers, often within highly competitive and lower-margin markets compared to pharmaceuticals.

Moreover, the infrastructure supporting this sector is still in its infancy. Unlike the pharmaceutical industry, which benefits from a mature and globally distributed network of CDMOs, the alternative protein space faces a critical bottleneck in access to scalable, food-grade fermentation facilities [21,25]. This limits the ability of startups to transition from laboratory-scale innovation to commercial production, particularly in emerging economies with weaker biomanufacturing capacity.

The intersection of food and biotechnology also introduces new governance and coordination issues. Startups and small and medium enterprises (SMEs) must navigate fragmented regulatory environments, cost-sensitive production models, and rapidly evolving consumer preferences while managing innovation cycles that are often shorter and more volatile than in biopharma. These dynamics affect how value is created and captured, and how capabilities are built and scaled across regions.

CDMOs thus play a particularly strategic role in this context, not merely as service providers, but as intermediary infrastructures capable of shaping the direction and inclusiveness of industrial development. In regions such as Latin America, their presence may determine whether local bio-innovations reach the market or remain locked at pilot scale.

As such, the alternative protein sector provides fertile ground for advancing theoretical understandings of intermediation, capability development, and value chain governance within emerging biomanufacturing ecosystems. The distinct features of this sector call for an expanded application of GVC theory, informed by insights from sustainability transitions, the circular bioeconomy, and institutional innovation [16].

Recent quantitative evidence reinforces both the scale of the opportunity and the scale-up challenge in fermentation-based proteins. Estimates suggest that fermented novel proteins could represent a USD 100–150 billion annual market by 2050, while precision and biomass fermentation ventures have attracted more than USD 4 billion in investment over the past five years. At the same time, scaling the sector may require more than USD 250 billion in cumulative capacity investment by 2050, while existing capacity to support scale-up remains limited. Recent industry evidence also indicates that contract manufacturing organizations often operate with margin expectations misaligned with food-sector economics, which can further constrain the availability of viable outsourced capacity. This gap between projected demand and available biomanufacturing infrastructure highlights the structural constraints faced by startups and reinforces the strategic role of CDMOs in enabling industrialization [20].

## 3. Methodological Approach

This article adopts a conceptual, theory-building approach grounded in an integrative literature review and abductive reasoning to develop an analytical framework that positions CDMOs within emerging fermentation-based protein global value chains (GVCs). The framework treats sustainability, circularity, and cleaner production as outcomes shaped by organizational, infrastructural, and governance arrangements, rather than as technological performance metrics. Following Torraco, the integrative review critically synthesizes and reorganizes a fragmented and interdisciplinary body of knowledge to generate new theoretical insights, an approach particularly suited to fields characterized by rapid technological change and evolving institutional settings.

The conceptual model was developed through an iterative and abductive analytical process, involving continuous engagement with both scholarly and practitioner-oriented sources. As emphasized by Shepherd and Suddaby [45], abductive reasoning in conceptual research enables the identification of novel theoretical relationships through iterative movement between empirical observations reported in the literature and established theoretical constructs. This process allowed insights from global value chain studies, bioeconomy research, sustainable production, and industrial biotechnology to be systematically integrated into a coherent analytical framework.

The analysis draws on a core corpus of more than 40 academic and industry sources published between 2017 and 2026, complemented by a selectively curated set of foundational texts that provide the theoretical backbone for the paper’s governance, coordination, intermediation, and upgrading constructs. The 2017–2026 window was adopted to capture the period in which fermentation-based alternative proteins and food-grade scale-up constraints became more explicitly articulated in both scholarly debates and industry-oriented evidence, including discussions on CDMO roles, manufacturing capacity, regulatory pathways, and investment-relevant scaling bottlenecks. Foundational texts were included only when they were directly mobilized to define and operationalize core analytical constructs, particularly GVC governance typologies and upgrading mechanisms, as well as innovation governance and intermediation insights related to value capture, industry architecture, and capability formation.

Academic sources were identified through targeted searches in major scholarly databases, including Scopus and Web of Science, using combinations of keywords related to CDMOs, fermentation-based biomanufacturing, alternative proteins, and global value chains. Google Scholar was used as a complementary tool to cross-check citations and retrieve relevant interdisciplinary and gray literature not consistently indexed in curated databases. Industry and policy reports were selected from recognized organizations, sectoral platforms, and institutional repositories, complemented by backward and forward citation tracking to capture influential and widely referenced contributions. Sources include peer-reviewed journal articles, technical and policy reports, and industry white papers. Selection was guided by relevance to four inter-related analytical dimensions: (i) CDMO functions and scale-up infrastructure; (ii) GVC governance structures and upgrading mechanisms; (iii) sustainability and circularity in fermentation-based biomanufacturing; and (iv) institutional and policy frameworks shaping bioeconomy development.

While the review does not aim at exhaustive coverage typical of systematic reviews, it follows a structured and transparent selection process consistent with PRISMA (Preferred Reporting Items for Systematic Reviews and Meta-Analyses) principles, adapted to support theory building and conceptual integration. To enhance transparency, the list of academic and industry sources informing the framework (Table S1), a detailed account of the search and selection process, and the PRISMA checklist (Table S3) are provided in the Supplementary Materials Table S1 includes the sources that directly informed the construction and interpretation of the framework’s core GVC dimensions, while other references cited in the text primarily serve to provide contextual background, empirical illustration, or sectoral detail.

The analytical process followed a structured reading and comparative synthesis strategy. Across the reviewed literature, recurrent patterns were identified regarding key stages of fermentation-based protein value chains, modes of interaction between CDMOs and client firms such as co-development, technology transfer, and contract manufacturing, governance configurations and coordination mechanisms shaping interfirm relationships, and upgrading trajectories at the process, product, functional, and intersectoral levels. Literature from adjacent sectors, particularly pharmaceuticals, food technology, and industrial biotechnology, was theoretically triangulated to examine how CDMOs mediate access to specialized knowledge, manufacturing infrastructure, regulatory capabilities, and control in fragmented and capital-intensive value chains.

Precision fermentation was used as a reference case in building the conceptual model because of its relatively high technological, regulatory, and coordination complexity, as highlighted in recent reviews of fermentation-based alternative proteins [11,46]. This makes the role of CDMOs more visible and easier to analyze, since activities such as strain engineering, downstream purification, regulatory compliance, and capital-intensive scale-up often rely on external capabilities and specialized infrastructure. Focusing on this setting allows the framework to capture coordination challenges, governance patterns, and upgrading dynamics in a more pronounced way.

Although the framework is anchored in precision fermentation, insights from the literature on microbial biomass and fungal protein production are used to extend its applicability to other fermentation pathways [47]. In these cases, upstream and downstream activities differ, with greater emphasis on strain selection, harvesting, and texturization rather than genetic engineering and purification. Even so, the underlying governance logic, coordination challenges, and upgrading mechanisms remain comparable.

This study does not involve empirical data collection and does not claim statistical generalizability. Instead, it offers a structured conceptual contribution derived from integrative synthesis and abductive reasoning. The outcome of this analytical process is an integrated conceptual framework that consolidates value chain stages, CDMO roles, governance modes, and upgrading mechanisms, presented in Section 5, and intended to inform future empirical research and policy design related to sustainable and inclusive scale-up models in fermentation-based biomanufacturing systems.

Potential selection biases were addressed through an iterative and abductive review process. Sources were selected based on their relevance to the analytical focus of the study, combining established academic contributions with industry reports that reflect ongoing developments in biomanufacturing. To avoid a single-domain perspective, the review brings together literature from global value chain governance, biomanufacturing scale-up, CDMO functions, and sustainability transitions. This enables triangulation across different bodies of knowledge and informs the development of the proposed framework.

## 4. Structuring Sustainable Fermentation Value Chains: The Role of CDMOs in Global Value Chain Integration

To contextualize the role of CDMOs in the alternative protein sector, it is first necessary to outline the core fermentation-based technologies that underpin this emerging industry. Biomass fermentation relies on microorganisms such as fungi, yeasts, or bacteria to produce protein-rich biomass that can be used directly as food ingredients. Precision fermentation, in turn, enables the production of specific functional proteins, such as milk caseins or egg ovalbumin, through genetically engineered microorganisms; it has been increasingly explored for the production of animal-identical dairy proteins [48]. Both approaches require sophisticated infrastructure for bioprocess optimization, scale-up, quality control, and regulatory compliance, functions that are typically beyond the reach of early-stage startups and are increasingly provided by CDMOs.

The capital expenditure required to build and operate fermentation facilities represents a major barrier to entry in this sector. Pilot-scale fermentation facilities may require investments in the range of one to three million US dollars, while commercial-scale precision fermentation plants can demand investments exceeding one hundred million US dollars [21]. These financial constraints reinforce the strategic importance of CDMOs as enabling actors that provide access to industrial infrastructure without the need for large upfront investments. By lowering entry barriers, CDMOs facilitate the transition from laboratory-scale innovation to commercial viability and contribute to the formation of globally dispersed fermentation-based value chains.

Against this technological and infrastructural backdrop, this study proposes a conceptual model that positions CDMOs as central enabling and coordinating actors within fermentation-based alternative protein global value chains. The model highlights how CDMOs interact with startups and established food industry players across different stages of the value chain, shaping scaling trajectories, knowledge transfer, governance structures, and sustainability outcomes. The following subsections elaborate on the key building blocks of this model.

### 4.1. CDMOs and Global Value Chains in Fermentation-Based Proteins

Fermentation-based protein production leverages microorganisms such as fungi, bacteria, microalgae, and yeasts to transform raw materials into nutritionally valuable food products. These processes can support sustainable protein production by reducing reliance on animal agriculture and by enabling the use of agricultural and industrial by-products as microbial feedstocks, thereby contributing to circular food systems [49,50]. Two dominant technological pathways structure this landscape: biomass fermentation, which produces protein-rich microbial biomass for direct consumption, and precision fermentation, which focuses on the biosynthesis of specific functional proteins through engineered microorganisms [10].

These technologies underpin emerging GVCs in which CDMOs play a critical role in enabling scale-up and commercialization. High capital intensity, technical complexity, and stringent regulatory requirements make access to specialized fermentation infrastructure essential. CDMOs provide fermentation capacity, downstream processing, bioprocess optimization, and regulatory support, allowing startups to overcome financial and operational barriers and accelerate their progression along the value chain [21].

**Figure 1:**
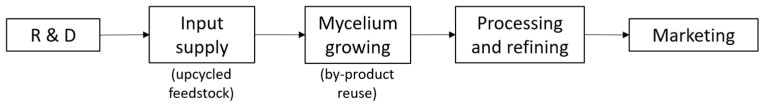
Illustrative value chain configuration in mycelium-based meat substitute production. Note: R&D = research and development. The “Input Supply” stage may include circular feedstocks such as brewery by-products, highlighting opportunities for resource valorization within fermentation-based protein systems.

For example, in the case of mycelium-based meat substitutes, produced through biomass fermentation, value chain activities typically include strain development, fermentation process optimization, cultivation, downstream processing to achieve meat-like textures, and subsequent distribution and marketing (Figure 1). An example of circularity in this context is the valorization of agro-industrial residues from the brewing industry, such as malt and fermentation byproducts, which can be repurposed as nutrient-rich feedstocks for fungal growth. These nutrient-rich by-products, when used as substrates, reduce waste, valorize existing supply chains, and have the potential to reduce the environmental footprint of microbial protein production [13].

Residual outputs from mycelium production—such as spent substrate or fermentation residues—can also be valorized through circular strategies, including use as animal feed, composting, or biogas generation, contributing to the environmental performance of fermentation-based protein systems. CDMOs, by offering flexible and modular bioprocessing services, are well-positioned to integrate such by-products into fermentation workflows, optimizing substrate formulations and ensuring compliance with quality and regulatory standards. This reinforces their role not only as scale-up enablers but also as operational catalysts of circular bioeconomy practices.

Early-stage startups tend to focus on research and small-scale production, while CDMOs facilitate the transition to larger-scale fermentation and processing by acting as intermediaries in knowledge development and transfer [51] (Figure 1). Similar dynamics characterize precision fermentation value chains, where CDMOs support strain engineering refinement, fermentation, recovery, and purification processes that require highly specialized infrastructure and expertise [10].

Collaboration between startups and CDMOs is shaped by varying degrees of knowledge codifiability. While certain aspects of production are governed by standardized operating procedures, others remain partially tacit and require close interaction and iterative learning during scale-up. These dynamics underscore the relevance of core GVC dimensions such as input-output structure, governance, and upgrading in shaping the evolution of fermentation-based alternative protein value chains.

Most alternative protein startups operate under business-to-business models, supplying ingredients to food manufacturers that finalize and market end products [21,27,52]. In this configuration, startups concentrate on technological development and early-stage production, while CDMOs provide access to fermentation capacity, regulatory expertise, and scale-up coordination. In doing so, they operate as innovation intermediaries that translate and broker capabilities among fragmented actors [53]. This division of labor contributes to value chain fragmentation, dispersing activities across multiple actors and locations, and increasing the need for coordination through formal contractual arrangements.

Collaboration is typically governed by a combination of business contracts, quality agreements, manufacturing agreements, technical task agreements, and regulatory documentation that define responsibilities, standardize processes, and ensure compliance with food safety and legal requirements [54]. While such arrangements enable scaling, they can also generate dependencies. Limited availability of food-grade CDMOs and competing demand for fermentation capacity may constrain startups’ operational flexibility and expose them to bottlenecks related to CDMO timelines and priorities [20,21].

At the same time, CDMOs often function as knowledge hubs, connecting startups with investors, technology partners, and established food companies. These network effects can enhance innovation and market access, but growing concentration among a small number of specialized CDMOs may also intensify competition for infrastructure access and reinforce power asymmetries within the value chain [7].

### 4.2. Geographical Scope and Spatial Implications

The spatial distribution of CDMOs plays a decisive role in shaping participation in fermentation-based alternative protein global value chains. CDMO capacity is heavily concentrated in technological hubs such as the Netherlands, Singapore, the United States, and parts of the European Union, where advanced infrastructure, skilled labor, supportive policies, and proximity to research institutions foster innovation and scaling [55,56]. These hubs benefit from knowledge spillovers and dense networks that reinforce their competitive advantage.

In contrast, regions such as Latin America, Africa, and parts of the Asia-Pacific face limited access to food-grade fermentation infrastructure, constraining local firms’ ability to upgrade and participate in high value-added segments of global value chains [21]. Geographic concentration thus reinforces global disparities, as firms in developed regions gain preferential access to critical infrastructure, while startups in emerging economies encounter higher barriers to scale-up [9,32].

Regulatory environments further shape geographical dynamics. Differences in approval pathways, such as the Generally Recognized as Safe process in the United States versus the Novel Foods Regulation in the European Union, influence time to market and production feasibility. CDMOs with regulatory expertise play a critical role in navigating these differences by supporting dossier preparation, compliance, and market entry [57].

Finally, variations in labor costs, energy prices, logistics, and access to feedstocks influence the location and competitiveness of CDMOs. Strategic positioning near consumer markets and innovation ecosystems enhances scalability and responsiveness, while proximity to universities and research centers strengthens collaboration and skill development. In addition, proximity to agro-industrial clusters—such as brewing or grain processing industries—can provide access to nutrient-rich by-products suitable for microbial fermentation, further supporting circular value chains. CDMOs that integrate local residue streams as inputs not only reduce production costs and environmental impact but also enable more inclusive forms of industrial development in underrepresented regions. In this context, the geographical configuration of CDMOs emerges as a key determinant of how sustainability benefits, industrial upgrading, and value capture are distributed across fermentation-based alternative protein global value chains.

### 4.3. Chain Governance

In fermentation-based alternative protein systems, chain governance is shaped by the technical complexity of scaling microbial fermentation and the institutional requirements for food-grade manufacturing [21,22]. Startups depend on CDMOs for infrastructure, regulatory compliance, and bioprocess optimization at scale, while CDMOs rely on startups’ proprietary strains, formulations, and market orientation to design effective production strategies [51,58,59]. This reciprocal interdependence creates governance structures that are neither fully integrated nor entirely transactional.

These interactions often manifest as modular governance, where each actor retains autonomy over its core competencies while coordinating through codified specifications and standardized processes, consistent with modular governance patterns described in GVC literature [29,30,58]. Startups usually remain limited to flask-level experiments or small-volume bioreactors, restricting scale-up validation. CDMOs overcome this bottleneck by supporting process scale-up and the production of pilot and commercial batches without requiring in-house industrial infrastructure.

Relational and hierarchical governance structures may be less prevalent in fermentation-based protein value chains. Relational governance typically arises when transactions are difficult to codify and rely on tacit knowledge exchange [29,30]. In fermentation systems, however, many bioprocess parameters—particularly at the scale-up stage—can be standardized and codified, reducing the need for intensive coordination. At the same time, the capital-intensive and infrastructure-dependent nature of biomanufacturing limits the availability of broadly capable suppliers. Within the GVC framework, this can be understood as a constraint on supplier capabilities which, in the presence of complex transactions, tends to favor captive rather than relational arrangements. Vertical integration remains less common due to the substantial investment required to internalize production capacity.

As a result, modular governance may emerge where capable partners and standardized interfaces are available, while captive forms tend to prevail under conditions of limited access to specialized infrastructure. While modular governance tends to prevail in later stages due to increased codifiability of bioprocess parameters, it remains underpinned by ongoing relational interactions, particularly in process optimization, troubleshooting, and scale-up adjustments, as widely documented in biomanufacturing and fermentation scale-up processes [51,58,59]. In this sense, codified protocols are often the outcome of prior relational learning processes, and their implementation frequently continues to rely on tacit knowledge exchange. This dynamic also raises potential concerns regarding knowledge leakage and capability erosion when critical process knowledge is co-developed with external manufacturing partners.

However, this modularity may constrain startups’ ability to appropriate value, especially when production capabilities and critical knowledge are externalized, reinforcing asymmetric value capture along the chain [7,59,60]. From a value capture perspective, externalizing critical manufacturing, scale-up, and regulatory capabilities does not eliminate value creation, but redistributes where value is captured along the chain. When specialized intermediaries control such capabilities, value capture tends to concentrate around those actors. Under modular production arrangements, this dynamic may become particularly salient when standardized platforms and interfaces facilitate coordination while simultaneously narrowing firms’ degrees of freedom for process experimentation and system-level differentiation. Importantly, this does not imply an inherent limitation of modular governance, but highlights how, under conditions of limited appropriability and capacity scarcity, modular architectures can amplify asymmetric bargaining positions and shape the distribution of innovation rents [59]. Moreover, modular setups may, under certain conditions, limit product differentiation, particularly when standardized CDMO platforms reduce opportunities for process customization and iterative experimentation.

Governance dynamics can shift toward captive structures when CDMO availability is limited, as in food-grade fermentation, where capacity is scarce [21]. In captive arrangements, startups become highly dependent on CDMOs, exposing them to power asymmetries, cost escalation, and delays in scale-up [7,30].

This dynamic may be intensified in geographically concentrated innovation ecosystems, where uneven access to complementary assets and specialized infrastructure shapes firms’ strategic positioning and opportunity sets [61]. Such asymmetries are exacerbated in regions with less developed biomanufacturing ecosystems, constraining equitable participation in global value chains. Studies of biopharmaceutical CDMOs indicate that outsourcing manufacturing and development functions is a rational response to high capital intensity and regulatory risk, but may also reinforce asymmetric dependencies when access to specialized infrastructure is limited [42].

Intellectual property (IP) concerns are another central feature of governance. CDMO partnerships often involve confidential formulations, strain libraries, and sensitive bioprocessing methods. Weak contractual protections may expose startups to intellectual property (IP) leakage, while asymmetric licensing terms can restrict their freedom to operate [62,63]. Startups with robust internal R&D and distinctive IP portfolios are better positioned to negotiate terms and strengthen bargaining power and contracting positions [64]. Strategic use of layered governance—balancing internal capabilities with selective outsourcing—can enhance both innovation and resilience.

Importantly, governance structures also affect the circularity potential of these value chains. As Schultz et al. [18] and Perdana et al. [19] argue, circular supply chain governance requires not only technical integration but also institutional mechanisms that enable waste valorization and inter-firm coordination. In fermentation-based systems, CDMOs operate within value chain configurations where circular strategies—such as the use of agricultural and agro-industrial by-products as fermentation feedstocks—may be integrated, depending on coordination, governance, and market conditions [14]. Where governance fails to support such integration—due to misaligned incentives or weak coordination—opportunities for sustainability upgrading may be lost.

Thus, the governance of fermentation-based protein chains must be understood not only in terms of power asymmetries and dependency, but also in terms of their capacity to support innovation, circularity, and inclusive industrial development. Lead firms, CDMOs, and institutional actors all play roles in shaping whether these systems remain extractive or evolve toward distributed, regenerative bioeconomy models. Balancing flexibility, IP protection, and circular integration is therefore critical for firms seeking to scale sustainably within fermentation-based global value chains.

### 4.4. Upgrading Mechanisms

In the fermentation-based alternative protein sector, product upgrading includes the development of formulations with enhanced nutritional and sensory profiles, such as improved texture and flavor of mycelium-based meat substitutes [20]. Process upgrading focuses on improving bioprocess efficiency, substrate utilization, and yield, especially by optimizing feedstock blends and fermentation parameters.

While CDMOs play a central role as process integrators and scale-up specialists in enabling process optimization, their technical expertise may both facilitate and constrain the adoption of circular feedstock strategies. On the one hand, advanced process engineering capabilities allow CDMOs to adapt fermentation parameters, optimize substrate utilization, and ensure consistency when using heterogeneous agro-industrial residues. On the other hand, strict process standardization, contamination risks, and regulatory requirements associated with food-grade production may limit the flexibility to incorporate variable or less-characterized feedstocks. In this context, the integration of circular inputs depends not only on their availability but also on the degree to which CDMO process architectures can accommodate variability without compromising efficiency and compliance. This tension reflects a broader trade-off between process efficiency and input flexibility, which becomes particularly critical in industrial-scale fermentation systems operating under strict quality and regulatory constraints.

These roles also have more direct implications for cleaner production. Process optimization and scale-up support can improve yields, reduce substrate losses, and lower energy or water use per unit of output by stabilizing fermentation performance and lowering input intensity at scale. The use of agro-industrial residues as feedstocks can reduce reliance on conventional inputs, redirect waste streams from lower-value uses, and, depending on the substrate and process, lower the overall environmental footprint, including GHG emissions and resource use. Regulatory and quality-support functions may also contribute indirectly by reducing batch failures, reprocessing, and material losses [14,40].

These effects occur through identifiable mechanisms. For instance, improved process control reduces input intensity per unit of output, while integrating circular feedstocks decreases reliance on primary agricultural inputs [47]. Regulatory support further limits batch failures, reducing waste and reprocessing requirements [42]. The framework does not assume that CDMOs automatically lead to cleaner production outcomes; instead, it highlights the organizational and technical mechanisms through which these outcomes can be enabled, shaped, or constrained.

Circular resource strategies can also be framed as process innovations, particularly when firms integrate the valorization of by-products into fermentation workflows. For instance, the use of brewery residues as fungal substrates reduces feedstock costs and promotes waste reduction, enhancing the environmental and economic performance of fermentation systems [13,14,25].

Functional upgrading occurs when startups advance beyond raw ingredient production to proprietary strain development, downstream refinement, and market-oriented activities such as branding and distribution [8]. Intersectoral upgrading is particularly relevant in this context as CDMOs often transfer knowledge and processes from pharmaceuticals or industrial biotechnology into the food domain, adapting infrastructure and capabilities to new regulatory and market conditions.

While partnerships with CDMOs enable access to infrastructure and expertise, overreliance on external partners may hinder internal capability development, limit intellectual property control, and constrain strategic differentiation [59]. In emerging regions, where CDMO capacity is limited, public research institutions can act as upgrading catalysts—but require investment to overcome scale and quality limitations [25].

Successful upgrading thus depends on both firm-level strategies and systemic enablers such as infrastructure, skilled labor, financing, and innovation networks. However, upgrading does not necessarily translate into more equitable value distribution, particularly under asymmetric governance structures [8,60]. Building localized technological capacity within systems of knowledge embedded in national innovation systems is critical for sustained competitiveness [65,66]. In contemporary biomanufacturing sectors, such capacities may increasingly involve intermediary infrastructures such as CDMOs, which support scale-up, compliance, and process learning.

### 4.5. Institutional Context

In fermentation-based protein systems, institutional environments shape the extent to which startups and CDMOs can contribute to sustainable innovation. Public policy and institutional design are often critical to enabling investment, coordination, and capability-building in emerging industries [33]. From this perspective, industrial policy operates not as a mechanism for selecting winners, but as a discovery-oriented process that reduces uncertainty and coordination failures through institutionalized public–private interaction. In such systems, policies that support shared pilot infrastructure and coordination platforms lower self-discovery costs and enable intermediaries such as CDMOs to emerge as mechanisms for capability diffusion and scaling. Moreover, from a GVC perspective, these conditions influence the feasibility of upgrading trajectories and the emergence of specialized intermediaries such as CDMOs [30].

The EAT–Lancet Commission highlights the urgency of transforming food systems within planetary boundaries [12]. Manufacturing infrastructures can support the scaling of novel production models when public investment and governance frameworks align economic incentives with circularity, biodiversity protection, and environmental performance. Institutional arrangements that integrate these objectives are therefore important for advancing cleaner production models consistent with food security goals. When embedded in regionally anchored innovation ecosystems and supported by public investment, CDMOs may contribute to sustainable scaling pathways.

These dynamics are especially consequential in under-resourced regions such as Latin America, Sub-Saharan Africa, and parts of Southeast Asia, where CDMO development is often constrained by uneven access to pilot and commercial-scale fermentation infrastructure, fragmented regulatory systems, limited patient capital, and weaker institutional support for cross-sectoral technology transfer. In such contexts, startups may face longer and more uncertain scale-up trajectories, greater dependence on foreign manufacturing partners, and reduced opportunities for local value capture and upgrading. At the same time, these regions may also present distinctive opportunities for more regionally embedded CDMO development, particularly where abundant agro-industrial by-products, emerging bioeconomy strategies, and public–private coordination efforts can support shared infrastructure, distributed manufacturing models, and stronger links between local innovation systems and production capacity. These conditions suggest that the geography of CDMO development is shaped not only by technological capability, but also by institutional capacity to coordinate infrastructure, regulation, and industrial learning. These dynamics suggest that participation in fermentation-based value chains depends not only on technological capabilities, but also on how infrastructure, regulatory frameworks, and financing conditions are aligned across regions.

National strategies can further promote circular supply chain governance by strengthening coordination across actors and enabling waste reduction and resource recirculation [18,19]. In fermentation-based systems, such approaches may also facilitate the valorization of agro-industrial residues and by-products as microbial feedstocks [50,67]. Policy instruments such as targeted subsidies for waste-reuse infrastructure or support for R&D in alternative feedstocks can enhance the economic viability of fermentation-based models while contributing to broader environmental objectives.

In under-resourced regions, shared fermentation facilities and bioprocessing clusters can help broaden access to innovation infrastructure. Public–private partnerships, alignment of food safety regulations, and support for cross-sectoral knowledge transfer may reduce entry barriers and strengthen participation in global biomanufacturing value chains.

## 5. A Conceptual Model for CDMO Integration into Fermentation-Based Protein Value Chains

**Figure 2:**
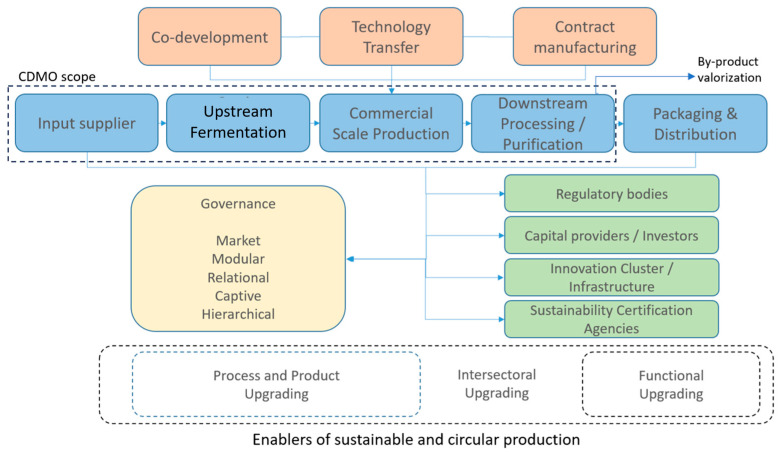
Conceptual model of CDMO integration in the fermentation-based protein global value chain.

Building on the preceding discussion, this section synthesizes GVC dimensions, CDMO functions, and sustainability considerations into an integrative conceptual model of fermentation-based protein value chains (Figure 2). The model conceptualizes CDMOs as intermediary infrastructures that shape governance arrangements, upgrading trajectories, and sustainability outcomes in biomass and precision fermentation systems. This positioning reflects the technical risks associated with microbial scale-up. In industrial biotechnology, fermentation processes exhibit scale-dependent uncertainty between laboratory, pilot, and industrial stages, making pilot-scale validation and specialized engineering capabilities particularly important [22].

The model places particular emphasis on precision fermentation due to its relatively high technological, regulatory, and capital intensity [10,11,46]. Drawing on GVC governance insights, such conditions are expected to increase coordination challenges and dependency risks when critical capabilities and biomanufacturing infrastructure are externalized. Precision fermentation, therefore, provides a useful case for examining how access to specialized infrastructure conditions firm behavior, governance modes, and upgrading opportunities. At the same time, evidence from microbial biomass and fungal protein systems supports the applicability of the model to fermentation pathways characterized by lower regulatory intensity [47,50,67].

It is important to highlight that CDMOs represent an emerging type of actor in global value chains for protein innovation, introducing organizational forms and enabling functions rarely central in conventional agri-food systems. Their presence may significantly reshape value chain structures and governance arrangements, acting as catalytic nodes that facilitate technological scaling and specialized biomanufacturing. This conceptualization adds novelty to GVC approaches applied to food systems by highlighting the role of enabling infrastructures in emerging biomanufacturing ecosystems.

### 5.1. Structure of the Model and CDMO Positioning

The model depicts fermentation-based protein value chains as a sequence of inter-related stages—input supply, strain development, fermentation, downstream processing, and B2B market integration—within which CDMOs operate as enabling and coordinating nodes. CDMOs provide access to fermentation capacity, regulatory expertise, and scale-up coordination. In doing so, they operate as innovation intermediaries, translating and brokering capabilities among fragmented actors [53]. In capital-intensive production environments, this intermediation can lower entry barriers and accelerate time-to-market for startups [21,38].

Drawing on recent reviews, the model distinguishes between two dominant fermentation pathways. In precision fermentation, value creation depends heavily on strain engineering, downstream recovery, and purification, as well as compliance with novel food and food safety regulations [10,46]. In biomass, the emphasis shifts toward strain selection, substrate optimization, harvesting, and texturization, often with greater scope for circular feedstock integration [47,67,68]. Despite these differences, both pathways rely on CDMOs to bridge laboratory-scale innovation and industrial-scale production.

### 5.2. Governance Configurations and Dependency Dynamics

From a GVC perspective, CDMO–startup interactions are governed by varying degrees of modular, relational, and captive governance [7]. Modular governance prevails when production specifications are highly codified and CDMO capacity is sufficiently available. However, in food-grade fermentation—particularly precision fermentation—limited infrastructure availability often shifts governance toward more captive arrangements, increasing dependency risks for startups [21].

Recent literature on alternative proteins highlights that regulatory complexity, capital intensity, and long scale-up timelines intensify coordination challenges and firms’ reliance on specialized infrastructure [69], which may, in turn, exacerbate governance asymmetries within fermentation-based value chains. In this context, CDMOs act not only as service providers but also as de facto gatekeepers of industrial scaling, influencing firms’ strategic options, learning trajectories, and value capture potential.

### 5.3. Upgrading Pathways and Circularity

Process upgrading in fermentation-based systems is strongly constrained by scale-dependent bioprocess behavior, which makes pilot-scale experimentation a prerequisite for industrial learning and cost reduction [22].

The model embeds upgrading mechanisms across four dimensions: process, product, functional, and intersectoral upgrading [8]. CDMOs enable process upgrading through bioprocess optimization, yield improvement, and integration of circular feedstocks, including agro-industrial residues and by-products [13,47,67]. Such practices enhance both environmental performance and cost efficiency, reinforcing the role of CDMOs in cleaner production pathways.

Product upgrading is reflected in the development of higher-functionality ingredients—such as dairy-identical proteins or structured fungal biomasses—supported by CDMO expertise in downstream processing and quality assurance [11,68]. Functional upgrading occurs when firms expand beyond ingredient supply toward branding, regulatory management, or downstream market integration, often facilitated by knowledge spillovers from CDMO partnerships.

Finally, intersectoral upgrading is central to the model: CDMOs transfer capabilities from pharmaceutical and industrial biotechnology value chains into food applications, adapting GMP-oriented infrastructures to food-grade requirements [38,43]. This cross-sectoral capability transfer is increasingly recognized as an enabling condition for scaling in fermentation-based alternative proteins [10,46].

### 5.4. Institutional and Geographical Embeddedness

The model explicitly incorporates institutional and geographical asymmetries shaping CDMO access and sustainability outcomes. Infrastructure concentration in a limited number of regions reinforces uneven participation in global value chains, particularly disadvantaging firms in emerging economies [21,25]. Reviews of alternative protein systems consistently emphasize that these bottlenecks constrain not only commercialization but also learning and upgrading [11,69].

Institutional actors, such as regulators, public research organizations, and policymakers, therefore play a critical role in mediating CDMO availability and governance conditions. By supporting shared fermentation infrastructure, circular feedstock valorization, and regulatory harmonization, policy frameworks can reduce dependency risks and foster more inclusive bioeconomy trajectories by shaping governance conditions, supporting circular integration, and enabling coordinated infrastructure development [16,18].

### 5.5. Implications and Research Directions: CDMOs and the Scaling of Sustainable Protein Systems

Alternative proteins present a credible pathway for transforming food systems through more sustainable and technologically advanced production models. In this context, CDMOs play a pivotal role by enabling access to fermentation infrastructure, process expertise, and regulatory capabilities. Yet, their limited availability—particularly outside established innovation hubs—continues to constrain sectoral development and restrict broader participation in global value chains.

This study extends GVC theory into an innovation-intensive and biotechnology-driven domain, highlighting how contractual and technological interdependencies shape emerging production systems. Rather than acting solely as service providers, CDMOs operate as infrastructural intermediaries that influence governance configurations, capability formation, and the distribution of upgrading opportunities across actors and regions.

From a practical standpoint, this implies differentiated strategic challenges. CDMOs must position themselves not only as providers of capacity but also as partners in process development and knowledge integration. Startups, in turn, face the dual task of leveraging external capabilities while progressively building internal competencies to avoid long-term dependency. For policymakers, the central challenge lies in strengthening enabling environments through targeted infrastructure investments, regulatory coordination, and support for distributed and regionally embedded innovation models.

While CDMO partnerships can accelerate time-to-market and reduce capital barriers, they also introduce risks related to dependency, coordination costs, and potential lock-in to specific technologies or providers. These tensions are not incidental but reflect the structural characteristics of capital-intensive and knowledge-driven value chains. As such, ensuring resilience and inclusivity requires careful alignment between firm strategies, governance arrangements, and institutional support mechanisms.

The role of CDMOs becomes particularly salient in the context of industrial policy and bioeconomy transitions. Their infrastructure and services form part of the institutional fabric through which innovation is scaled. When supported by coordinated public and private investment, CDMOs can catalyze systemic change by reducing uncertainty and enabling experimentation in emerging sectors. At the same time, if infrastructure remains geographically concentrated, these same dynamics may reinforce existing asymmetries in access to production capacity and technological capabilities.

Addressing these imbalances requires a combination of policy instruments and strategic adaptation. Public investment in pilot and commercial-scale fermentation infrastructure, alongside mechanisms for technology transfer and capability development, can lower entry barriers and expand participation. In parallel, the adaptation of existing facilities—such as those in brewing or related fermentation industries—offers a pragmatic pathway for scaling in resource-constrained environments. These approaches are particularly relevant for regions seeking to integrate into higher value-added segments of bio-based production systems.

At the firm level, diversified engagement with CDMOs across different stages of development—from R&D to commercialization—can enhance flexibility while mitigating risk. However, this must be complemented by deliberate strategies for knowledge retention, IP management, and gradual internalization of critical capabilities. The balance between externalization and internalization becomes a central strategic question in shaping long-term positioning within the value chain.

The framework developed in this article also points to several directions for future research. One key avenue is to examine how CDMO–startup relationships evolve across governance configurations under varying conditions of codifiability, infrastructure scarcity, and regulatory complexity. In particular, empirical studies could clarify when modular arrangements prevail, when more captive dynamics emerge, and how relational interactions continue to shape process optimization and scale-up learning even in highly codified environments.

To support such empirical work, the framework can be operationalized at different levels, including firm relationships, value chain segments, or regional biomanufacturing ecosystems. Governance configurations can be examined through the degree of codifiability, contractual formalization, dependence on external manufacturing capacity, and the balance between modular, relational, and captive interactions. Upgrading trajectories may be assessed through observable changes in process efficiency, product functionality, internal capability development, movement into higher value-added activities, and cross-sectoral capability transfer. Institutional and geographical embeddedness can be explored through indicators such as access to pilot and commercial infrastructure, regulatory support, public policy instruments, financing conditions, and the presence of innovation networks or shared facilities. These dimensions can be investigated through comparative case studies, multi-case designs, interview-based process tracing, or mixed-method approaches combining qualitative evidence with firm- or region-level indicators.

A second line of inquiry concerns upgrading and value capture. Future research should explore how access to CDMOs influences process, product, functional, and intersectoral upgrading trajectories, and under what conditions outsourcing supports capability development rather than erosion. This includes examining how circular feedstock strategies interact with process standardization, regulatory constraints, and quality-control requirements in industrial-scale fermentation systems.

Finally, greater attention is needed to the institutional and geographical embeddedness of CDMO development. Comparative research across regions can shed light on how policy frameworks, innovation systems, and infrastructure availability shape participation in global value chains. This is particularly critical in under-resourced regions, where unequal access to biomanufacturing infrastructure may limit both industrial upgrading and the realization of cleaner production outcomes.

Taken together, these implications reinforce the central argument of the paper: CDMOs are not peripheral actors but key structural elements in the organization and scaling of fermentation-based protein systems. Their role in mediating access to infrastructure, knowledge, and coordination mechanisms places them at the core of both technological and institutional transformations in emerging bio-based industries.

## 6. Conclusions

This study develops a conceptual framework to examine the role of CDMOs in shaping global value chains for fermentation-based proteins. By positioning CDMOs as intermediary infrastructures that influence governance structures, capability development, and innovation scaling, the study highlights their dual role as enablers of cleaner production and potential sources of dependency and asymmetry. Drawing on Global Value Chain theory and an integrative, abductive synthesis, the analysis shows how CDMOs mediate access to critical infrastructure, technical expertise, and institutional environments—thereby conditioning how sustainability and circularity objectives are translated into industrial practice in emerging biomanufacturing systems.

The framework contributes to GVC scholarship by extending its application to innovation-intensive sectors characterized by fragmented infrastructure, technological interdependence, and rapid industrial evolution. At the same time, it speaks directly to debates on cleaner production by illustrating how organizational and infrastructural configurations shape environmental performance beyond the characteristics of individual technologies. The analysis offers practical insights for policymakers and industry actors seeking to foster more inclusive and regionally distributed fermentation capacity, particularly in under-resourced regions where infrastructure and regulatory gaps persist. The findings suggest that CDMO engagement must be strategically coordinated to avoid reinforcing geographic disparities, constraining value capture, and limiting firms’ innovation autonomy.

The applicability of the proposed framework is, however, conditioned by specific characteristics of fermentation-based biomanufacturing systems. In particular, it assumes the presence of high capital intensity, dependence on specialized infrastructure, and the relevance of externalized scale-up capabilities. In contexts where production processes are less infrastructure-constrained, more easily internalized, or less dependent on regulatory compliance and process standardization, the centrality of CDMOs as coordinating intermediaries may be reduced.

Supporting sustainable transitions in protein production therefore requires coordinated public–private action to expand shared biomanufacturing infrastructure, invest in CDMO-related capability development, and institutionalize circular supply chain governance. Policy measures that promote by-product valorization, cross-sectoral learning, and broader participation in bioeconomy value chains can help align industrial scaling with cleaner production objectives. The framework is most applicable to contexts characterized by high capital intensity, dependence on specialized biomanufacturing infrastructure, and the externalization of scale-up capabilities. Given its abductive and integrative nature, the framework reflects patterns identified across a selected body of literature. As such, certain emerging configurations, alternative organizational models, or region-specific dynamics may be underrepresented. The framework also assumes that governance, upgrading, and institutional conditions interact in relatively structured ways, which may not fully capture non-linear or path-dependent dynamics observed in early-stage innovation ecosystems.

Future research should refine and empirically test the proposed framework through comparative and case-based studies, examining CDMO dynamics across geographies, technologies, and governance configurations. Further work is also needed to assess the extent to which the framework applies to other bio-based or deep-tech sectors, particularly those with different cost structures, regulatory regimes, or degrees of technological modularity. Such efforts are important for advancing more inclusive and environmentally sustainable food system and for strengthening the institutional foundations of cleaner production in emerging biomanufacturing sectors.

## Data Availability

This study is based on an integrative review of published academic and industry sources. No primary datasets were generated or analyzed. All sources used are cited in the article and Supplementary Materials.

## Supplementary Materials

The following supporting information can be downloaded at: https://www.mdpi.com/article/10.3390/foods15081341/s1, Table S1: Representative literature informing the analytical dimensions of the conceptual framework, ordered by year of publication; Table S2: Keyword groups used in the literature search; Table S3: PRISMA 2020 checklist; Figure S1: PRISMA-informed flow diagram of the literature identification and selection process for the integrative review. Adapted for integrative conceptual review.

## Abbreviations

The following abbreviations are used in this manuscript:

B2B	Business-to-Business
CAGR	Compound Annual Growth Rate
CDMOs	Contract Development and Manufacturing Organizations
GHG	Greenhouse Gas
GMP	Good Manufacturing Practices
GVC	Global Value Chain
IP	Intellectual Property
R&D	Research and Development
SDGs	Sustainable Development Goals
SMEs	Small and Medium Enterprises
