# Supplementary material for: Infrastructure for Sustainable Protein Innovation: A Global Value Chain Framework for CDMOs in Fermentation-Based Biomanufacturing
Source: Foods. 2026 Apr 13;15(8):1341. doi: 10.3390/foods15081341 (PMC13114431; doi:10.3390/foods15081341)
Supplement: Supplementary file 1 [file foods-15-01341-s001.zip › foods-4213523-supplementary.pdf]

Supplementary Materials

Table S1 – Representative literature informing the analytical dimensions of the conceptual framework, ordered by year of publication

Note: This table presents the literature base that informed the abductive development of the conceptual framework. Inclusion reflects analytical relevance rather than exhaustiveness. The analytical dimensions reported do not reproduce the full scope of each contribution, but explicitly identify the main conceptual elements mobilized in the abductive construction of the proposed framework.

Author(s)	Year	Main focus	Framework-relevant analytical dimensions
Bell & Albu	1999	Learning systems and industrial transformation	Capability building and systemic learning as foundations for industrial transformation and upgrading trajectories
Kaplinsky	2000	Rents, power and value distribution in GVCs	Power asymmetries and rent distribution shaping value capture across global value chains
Humphrey & Schmitz	2002	Upgrading trajectories in GVCs	Typologies of upgrading paths explaining how firms reposition within global value chains. Process, product, functional, intersectoral upgrading
Rodrik	2004	Industrial policy as a discovery process addressing coordination and information failures	Institutional context; coordination and information failures shaping capability development and viable upgrading trajectorie
Gereffi, Humphrey & Sturgeon	2005	Governance typologies in global value chains	GVC governance configurations, defining coordination, control, and upgrading opportunities within global value chains
Jacobides, Knudsen & Augier	2006	Value creation and appropriation shaped by industry and value chain architecture	Governance; Structural constraints on value capture (arising from modularity and interdependence)
Howells	2006	Intermediation and the role of intermediaries in innovation systems	Innovation intermediation through brokering, translation,

Author(s)	Year	Main focus	Framework-relevant analytical dimensions
			and coordination of capabilities across fragmented actors
Pisano	2006	Appropriability regimes and the conditions under which innovators capture value from innovation	Governance; value capture shaped by reliance on critical complementary assets
Wadhwa et al.	2008	Global fragmentation of pharmaceutical innovation and the emergence of specialized intermediaries in global value chains	Input–output structure; contract manufacturing; dependency dynamics; governance; functional upgrading through intermediaries
Haakonsson	2009	Pharmaceutical GVC governance	Differentiated governance structures; functional fragmentation; outsourcing and control
Pietrobelli & Rabellotti	2011	Global value chains and local development	Learning, upgrading, institutional embeddedness
D'Amato et al.	2017	Conceptual comparison of green, circular, and bioeconomy approaches to sustainability transitions	Circularity as a systemic outcome contingent on value chain configuration, institutions, and governance arrangements
Crater & Lievense	2018	Scale-up of industrial microbial processes	Scale-up constraints; pilot-scale validation; infrastructure intensity
Holgersson, Granstrand, & Bogers	2018	Evolution of intellectual property strategies and appropriability regimes in innovation ecosystems	Governance and value capture; appropriability regimes shaping bargaining power and coordination
Gereffi & Fernandez-Stark	2016	Conceptual and methodological foundations of GVC analysis	Foundational concepts and analytical tools for examining governance, structures, upgrading trajectories, and the institutional domain in GVCs
Lewandowski et al.	2019	Biobased value chains and systemic coordination in the bioeconomy	System-level value chain perspective; institutional coordination and policy support in bio-based production systems; value-chain-based

Author(s)	Year	Main focus	Framework-relevant analytical dimensions
			analysis of bioeconomy transitions
Gregg et al.	2020	Review of bio-residual valorization pathways and value chain configurations in support of a circular bioeconomy	Grounding for conceptualizing circularity as an outcome contingent on value chain configuration, governance structures, and institutional conditions
Ayoub et al.	2025	Bioeconomy and GVC integration: transition from niches to global value chains through collaborative strategies	Inter-firm collaboration and value chain reconfiguration, enabling niche-to-GVC integration; operational coordination; contractual arrangements
Schultz et al.	2021	Circular supply chain governance and horizontal coordination	Horizontal governance; collective action; coordination mechanisms;
Battle et al.	2023	Mapping of the fermentation-based alternative protein sector and its structure	Input–output structure; identification of intermediaries and infrastructure bottlenecks
Perdana et al.	2023	Circular supply chain governance in agri-food systems	Governance and coordination mechanisms enabling circularity and by-product valorization
Kårlund et al.	2020	Fermentation improving nutritional quality of alternative protein sources	Process-dependent nutritional and sustainability outcomes; fermentation benefits contingent on controlled production and application context
Molfetta et al.	2022	Fermentation pathways for alternative proteins	Input–output structure; technological differentiation across fermentation pathways
Bajić et al.	2022	Industrial SCP production from residues	Supports relevance of industrial fermentation and circular bioeconomy inputs; circular feedstocks; process upgrading

Author(s)	Year	Main focus	Framework-relevant analytical dimensions
Good Food Institute	2023	Fermentation processes, scale-up, and manufacturing capacity	Fermentation processes; infrastructure bottlenecks; CDMO relevance
Wang et al.	2023	Microbial protein production pathways	Process- and substrate-dependent sustainability outcomes; need for coordinated production and processing choices
Friedman	2024	Global biomanufacturing trends and CDMO industry dynamics	Infrastructure constraints; CDMOs as intermediaries coordinating access to specialized manufacturing capabilities
de Mello et al.	2024	Bioreactor scale-up engineering	Scale-up infrastructure; CDMOs coordinating outsourced production and capacity access
Többen et al.	2024	Bioeconomy transition along global supply chains	Cross-border trade-offs; uneven SDG impacts; import-dependency vs food-security tensions
Knychala et al.	2024	Precision fermentation technologies	Technological and regulatory complexity of precision fermentation; scale-dependent process shaping coordination and infrastructure requirements
Malila et al.	2024	Barriers to alternative protein scaling	Capital intensity and regulatory fragmentation as barriers constraining coordination, learning, and upgrading in alternative protein value chains
Mac Clay et al.	2024	Technology-driven transformations in agri-food global value chains	Industry structure shaping technological trajectories; separation between innovation and product supply chains; governance effects on scale-up and technology selection

Author(s)	Year	Main focus	Framework-relevant analytical dimensions
Meng & Zhe	2024	Comparative development of CDMOs across regions (pharmaceutical sector)	Institutional and policy-driven emergence of CDMOs; infrastructure development trajectories; role of regulation and state support in shaping scale-up intermediaries
Ferrero et al.	2024	Fungal biomass fermentation using by-products	Technical feasibility of circular feedstock integration via fungal biomass fermentation; process-level upgrading potential conditional on substrate characteristics
Juhász, Lane & Rodrik	2024	Reframing industrial policy through coordination, learning, and governance	Infrastructure as a selective enabling input; coordination failures; learning and experimentation; governance conditions shaping industrial scaling
Choi et al.	2024	Microbial foods from sustainable feedstocks	Expansion of the technological frontier of microbial foods through alternative carbon and energy feedstocks; implications for scale-up feasibility and resource efficiency
Verma et al.	2025	Precision fermentation opportunities and challenges	Complex multi-stage process architecture and regulatory intensity in precision fermentation, reinforcing scale-up barriers and the need for specialized production infrastructure
Boschma, R. et al.	2025	Interaction between global value chains and local capabilities in shaping regional economic complexity	Complementarity between GVC participation and localized productive capabilities enabling upgrading and innovation
Aminetzah et al.	2025	Industry assessment of fermentation-derived food ingredients and structural scale-up challenges	Input–output structure; infrastructure constraints and scale-up challenges in emerging fermentation value chains

Author(s)	Year	Main focus	Framework-relevant analytical dimensions
Pinheiro et al.	2026	Fermentation pathways in cellular agriculture	Technological differentiation across alternative protein pathways highlighting multi-dimensional viability conditions

Account of the search and selection process

The literature search aimed to identify contributions addressing fermentation-derived proteins, biomanufacturing infrastructure, value chain governance, and sustainability-oriented production systems. Searches were conducted in Scopus, Web of Science, and Google Scholar, complemented by targeted screening of relevant industry and policy reports. The search strategy combined keywords related to four thematic domains (Table A):

— fermentation-based proteins

— biomanufacturing infrastructure

— value chain governance and industrial scaling

— sustainability and cleaner production

Boolean operators were used to combine these keyword groups in order to identify literature addressing the intersection of technological development, production organization, and sustainability outcomes. Search strings were iteratively refined during the review process to ensure conceptual relevance to fermentation-based protein systems while capturing interdisciplinary perspectives from global value chain re-search, sustainability transitions, and circular bioeconomy studies.

**Conceptual domain**	**Keywords**
Fermentation-based proteins	("precision fermentation" OR "biomass fermentation" OR "fermentation-based protein*" OR "fermentation-derived protein*" OR "microbial protein*" OR "single-cell protein*" OR "alternative protein*" OR "cellular agriculture")
AND	
Biomanufacturing infrastructure	("biomanufacturing" OR "industrial fermentation" OR "fermentation capacity" OR "bioprocessing" OR "contract development and manufacturing organization*" OR CDMO*)
AND	
Value chain governance and scaling	("global value chain*" OR "value chain governance" OR "production network*" OR intermediar* OR "innovation scaling" OR "industrial scaling")
AND	
Sustainability and cleaner production	("sustainab*" OR "cleaner production" OR "circular bioeconomy" OR "circular economy" OR "resource efficiency" OR "environmental performance" OR "sustainability transition*")

Note: Shorter keyword combinations derived from these categories were used in Google Scholar to capture additional academic publications and grey literature.

Table S2. Keyword groups used in the literature search.

The literature identification and selection process followed a PRISMA-informed approach adapted for integrative conceptual reviews (Figure 1).

Figure S1 - PRISMA-informed flow diagram of the literature identification and selection process for the integrative review. Adapted for integrative conceptual review.

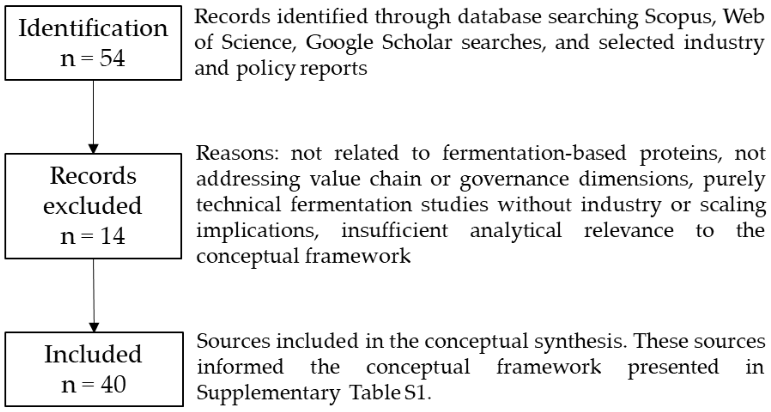

A total of 54 records were initially identified through database searches and additional sources, including academic publications, industry reports, and policy documents. Following title and abstract screening, 14 records were excluded because they were not related to fermentation-based proteins, did not address value chain or governance dimensions, focused exclusively on technical fermentation research without industry or scale-up implications, or lacked analytical relevance to the conceptual framework. After screening, 40 sources were included in the analytical corpus and in-formed the abductive development of the conceptual framework examining the role of Contract Development and Manufacturing Organizations (CDMOs) as infrastructural intermediaries in fermentation-based protein value chains.

Table S3, PRISMA 2020 checklist

This checklist reports the PRISMA 2020 items addressed in the manuscript. Items not applicable correspond to methodological elements specific to statistical systematic reviews and meta-analyses and therefore do not apply to the integrative conceptual review design adopted in this study.

PRISMA 2020 Checklist, Adapted for Integrative Conceptual Review

Section and topic	Item	Checklist item	Location where item is reported
Title	1	Identify the report as a systematic review	Title and Abstract (integrative literature review approach described)
Abstract	2	See PRISMA 2020 for Abstracts checklist	Abstract
Introduction	3	Describe the rationale for the review	Introduction
	4	Provide an explicit statement of the objective(s)	Introduction
Methods	5	Specify inclusion and exclusion criteria	Methodological Approach
	6	Specify databases, websites, organisations and other sources searched	Methodological Approach
	7	Present search strategies	Methodological Approach
	8	Describe study selection process	Methodological Approach
	9	Describe data collection process	Not applicable for conceptual integrative review
	10a	Outcomes for which data were sought	Not applicable – conceptual synthesis
	10b	Other variables collected	Not applicable – conceptual synthesis
	11	Risk of bias assessment	Not applicable for conceptual integrative review
	12	Effect measures	Not applicable

Section and topic	Item	Checklist item	Location where item is reported
	13a	Describe processes used to decide which studies were eligible	Methodological Approach
	13b	Methods used to prepare data for synthesis	Not applicable
	13c	Methods used to tabulate or visually display results	Supplementary Table S1
	13d	Methods used to synthesize results	Methodological Approach
	13e	Methods to explore heterogeneity	Not applicable
	13f	Sensitivity analyses	Not applicable
	14	Reporting bias assessment	Not applicable
	15	Certainty assessment	Not applicable
Results	16a	Results of search and selection process	Supplementary Figure S1
	16b	Excluded studies with reasons	Supplementary Figure S1
	17	Characteristics of included studies	Supplementary Table S1
	18	Risk of bias in studies	Not applicable
	19	Results of individual studies	Not applicable – conceptual synthesis
Results of syntheses	20a	Summary of study characteristics contributing to synthesis	Supplementary Table S1
	20b	Statistical synthesis results	Not applicable
	20c	Investigation of heterogeneity	Not applicable
	20d	Sensitivity analyses	Not applicable
Reporting biases	21	Reporting bias assessment	Not applicable
Certainty of evidence	22	Certainty assessment	Not applicable
Discussion	23a	Interpretation of results	Discussion

Section and topic	Item	Checklist item	Location where item is reported
	23b	Limitations of the evidence	Discussion
	23c	Limitations of the review process	Discussion
	23d	Implications for research and policy	Discussion
Other information	24a	Registration information	Review not registered
	24b	Protocol availability	No protocol prepared
	24c	Amendments to protocol	Not applicable
	25	Sources of support	Funding / Acknowledgements
	26	Competing interests	Conflict of Interest statement
	27	Availability of materials	Supplementary Materials
